# Sigmoid volvulus in children: a case report

**DOI:** 10.1186/s13256-017-1440-y

**Published:** 2017-11-07

**Authors:** Fayza Haider, Nabeel Al Asheeri, Barrak Ayoub, Eizat Abrar, Jawad Khamis, Hasan Isa, Husain Nasser, Fatima Al Hashimi

**Affiliations:** 1 Pediatric Surgery Unit-Department of Surgery, Salmaniya Medical Complex, P.O. Box 12, Manama, Bahrain; 2 Department of Medicine, Salmaniya Medical Complex, Manama, Bahrain; 3 Department of Pediatrics, Salmaniya Medical Complex, Manama, Bahrain; 4 Department of Radiology, Salmaniya Medical Complex, Manama, Bahrain; 5 Department of Pathology, Salmaniya Medical Complex, Manama, Bahrain; 6 Salmaniya Medical Complex, 12, Manama, Bahrain

**Keywords:** Case report, Sigmoid volvulus, Children, Adolescents, Decompression, Endoscopy, Sigmoidectomy, Primary anastomosis, Coffee bean sign

## Abstract

**Background:**

Sigmoid volvulus is frequently reported in the “volvulus belt” (Middle East, Africa, the Indian subcontinent, Turkey, and South America) and is the third leading cause of large bowel obstruction in North America.

It is an uncommon problem in children and adolescents, and is rarely considered a diagnosis in this group. A high index of suspicion is necessary to diagnose sigmoid volvulus in children.

**Case presentation:**

We present a 13-year-old Arabian girl who came with features suggestive of intestinal obstruction. Plain abdominal film revealed classic omega (coffee bean) sign of sigmoid volvulus. The volvulus was successfully decompressed by means of a rectal tube in our emergency department. The next day during the same admission the volvulus recurred and was successfully decompressed by endoscopy. She was discharged home on her parents’ request; she presented again 1 month later. This time the volvulus could not be decompressed non-operatively, so she underwent sigmoidectomy with primary anastomosis. Postoperatively she developed paralytic ileus that resolved after 10 days. Following that she did well and was discharged home. She is still free of symptoms 1 year after the resection.

**Conclusions:**

Sigmoid volvulus is an uncommon problem in children and adolescents, and is rarely considered a diagnosis in this group as a cause of intestinal obstruction. Pediatric surgeons should maintain a high index of suspicion, in order not to miss this important diagnosis, as any delay in instituting treatment has a devastating effect on morbidity as well as mortality. Early diagnosis and prompt treatment confer an excellent prognosis.

## Background

From *The Papyrus Ebers*: “If he doesn’t evacuate for a twist in the bowel and the phlegm does not find a way out then it shall rot in the belly” [1].

Sigmoid volvulus is frequently reported in the “volvulus belt” (Middle East, Africa, the Indian subcontinent, Turkey, and South America) and is the third leading cause of large bowel obstruction in North America [2]. It is an uncommon problem in children and adolescents, and is rarely considered a diagnosis in this group [3]. Sigmoid volvulus is an exceptionally rare and potentially life-threatening condition in the pediatric age group. A high index of suspicion is necessary to reach a diagnosis and avoid morbidity and mortality.

## Case presentation

**Fig. 1:**
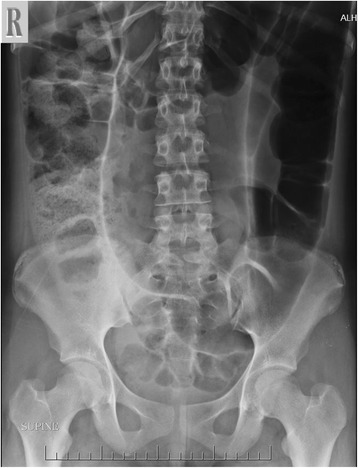
Plain radiograph of the abdomen of a 13-year-old girl showing hugely dilated loop of bowel, arising from her pelvis. It has the appearance of a coffee bean. Frimann Dahl’s sign is positive with three dense lines converging toward site of obstruction. Her rectum still contains air

**Fig. 2:**
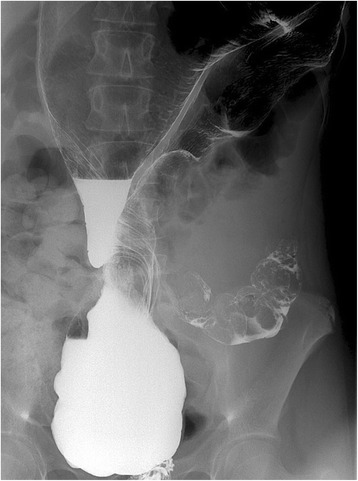
Contrast enema of a 13-year-old girl showing a markedly dilated sigmoid colon with the contrast medium passing to the sigmoid colon, which indicates incomplete obstruction. The twist of the colon is clearly seen

**Fig. 3:**
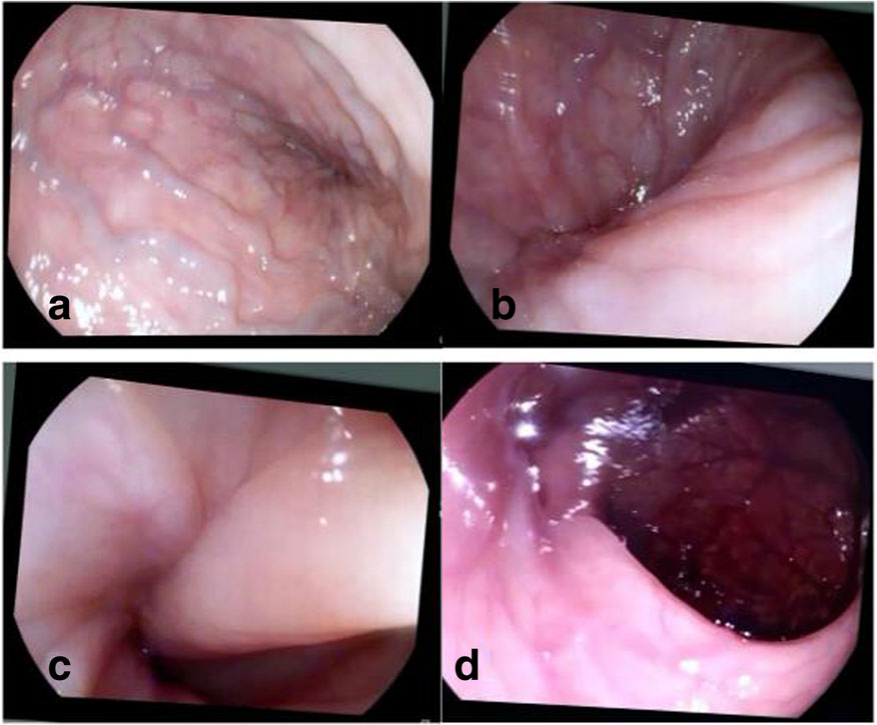
Endoscopic reduction of sigmoid volvulus. **a** Congested rectal veins distal to the twist; **b** the twist is visualized; **c** the volvulus opens slowly. **d** Full reduction achieved

**Fig. 4:**
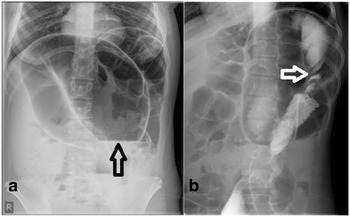
**a** Plain erect abdominal film of a 13-year-old girl showing markedly dilated sigmoid colon with coffee bean sign and air fluid level (*arrow*). **b** The contrast medium passed to the sigmoid colon and the twist is clearly seen (*arrow*)

**Fig. 5:**
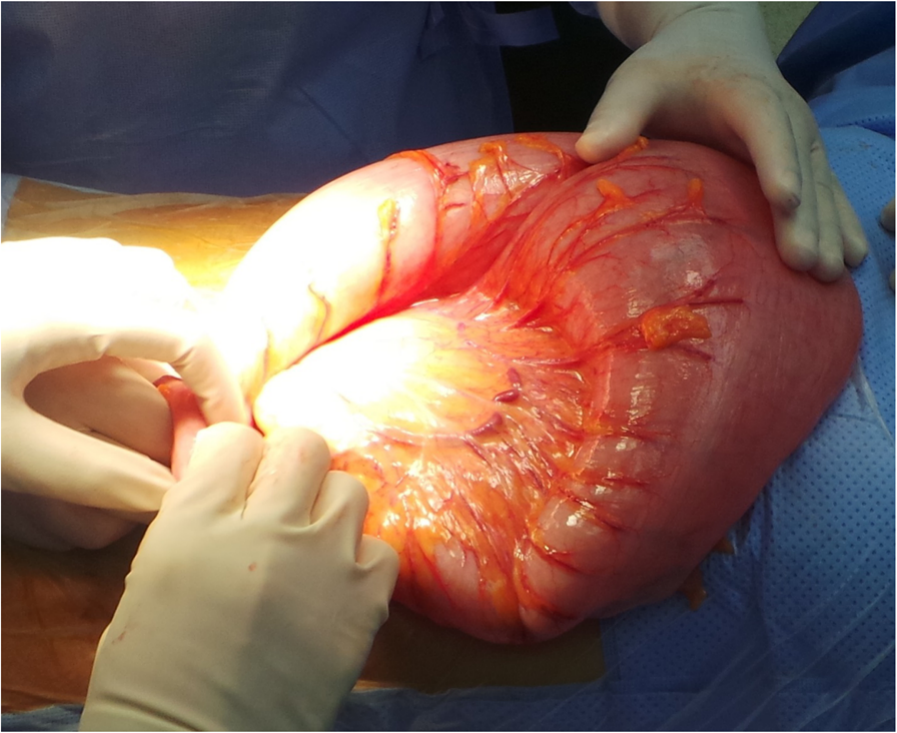
Intraoperative picture of a massively dilated sigmoid colon with 360^o^ twist

**Fig. 6:**
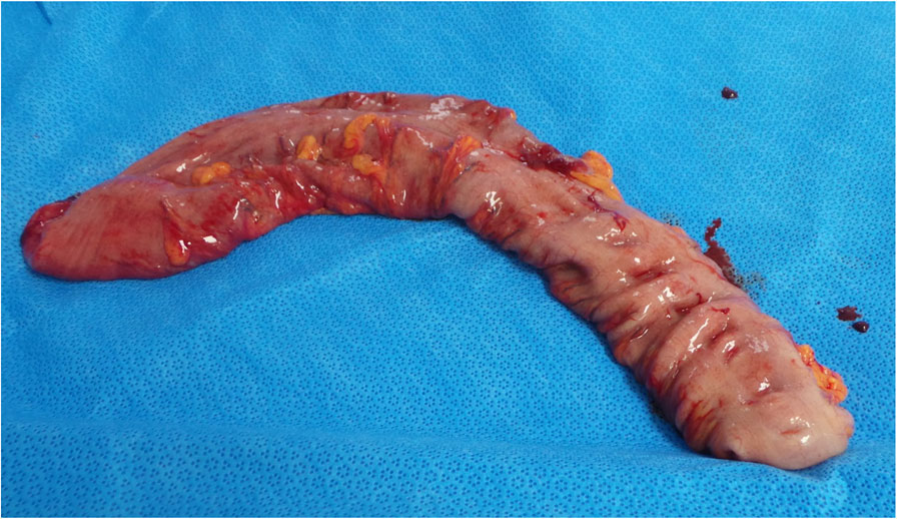
Resected sigmoid colon measuring 30 × 6 × 4 cm

**Fig. 7:**
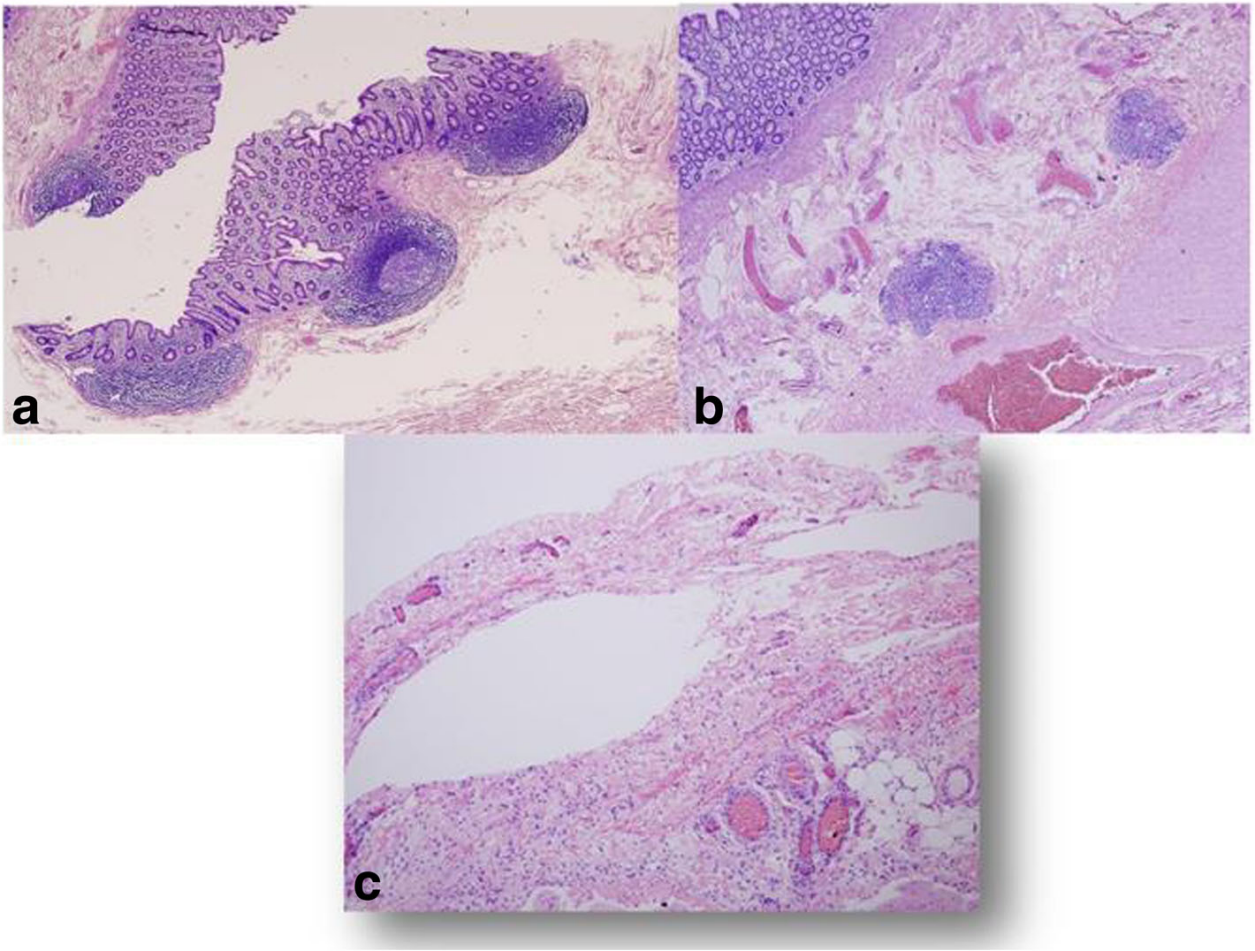
Histopathology of resected sigmoid. **a** Both resection margins showed a preserved architecture, with no features of ischemia and both the mucosal lining and muscular wall were viable. **b** The mucosal lining from the discolored areas showed mild chronic inflammation. The lamina propria has congested blood vessels overlying a viable but hypertrophic muscular coat. The features may be linked to acute on chronic obstruction. **c** There were visible ganglion cells and hypertrophic nerve trunks in the muscularis propria

Our case is a 13-year-old Arabian girl who presented to our institution referred from a periphery center with 3 days’ history of colicky abdominal pain, vomiting, and constipation. She did not report any previous episodes of constipation. She was reviewed at our emergency department and was found to have a distended tympanic abdomen that was soft all over with no tenderness. Bowel sounds were sluggish and a digital rectal examination revealed an empty rectum. A plain abdominal film showed a hugely dilated loop of bowel, arising from her pelvis, which had the appearance of a coffee bean (Fig. 1). The plain abdominal radiograph confirmed the presence of a sigmoid volvulus. A rectal tube was inserted carefully to decompress the volvulus which succeeded and gave her immediate relief from her symptoms. She was admitted to our pediatric surgical ward and had a nasogastric tube inserted and was started on intravenously administered antibiotics. The next day she was asymptomatic but a contrast enema showed the twist to be evident with an incomplete obstruction (Fig. 2). Due to the findings on contrast enema she went for endoscopic decompression of the volvulus under general anesthesia. The twist was causing venous congestion and was successfully reduced (Fig. 3). A rectal tube was left in place post endoscopic reduction for 24 hours. She was asymptomatic thereafter but her parents requested discharge and they were granted their wish as they wanted to travel to their country. In her country she was investigated and was advised conservative management and observation. A month after her previous attack, and after she had returned to Bahrain from her native country, she presented with the same signs and symptoms that she had in our institution with the same radiological findings but this time the volvulus could not be decompressed by a rectal tube at the emergency department or by endoscopy (Fig. 4). Her parents were advised that she should undergo surgery to which they consented. As this was considered an emergency, her bowel was not prepared. She was taken to an operative room 24 hours after the failed endoscopy reduction. She underwent laparotomy with the finding of the sigmoid occupying her whole abdominal cavity and was found on delivery to be hugely dilated with a 360^o^ twist. There was no evidence of gangrene of the bowel and the rest of her colon was healthy and normal (Fig. 5). We performed a sigmoidectomy with primary anastomosis (Fig. 6). The resected sigmoid showed no features of ischemia, but evident features of chronic inflammation and abundant ganglia (Fig. 7). Postoperatively she had paralytic ileus that resolved after 10 days. Following that she did well and was discharged home on the 12th postoperative day. She is still free of symptoms 1 year after her resection.

## Discussion

Although volvulus in adults is common in Asian populations, more cases in pediatric practice have been reported from the West, including North America. Among children several predisposing factors have been identified. Hirschsprung’s disease has been implicated in both transverse colonic as well as sigmoid volvulus [4]. This was ruled out in our patient as a cause for her sigmoid volvulus by the findings of abundant ganglia shown in biopsy section. Chronic constipation is another advocated factor for the development of sigmoid volvulus, but our patient and her family denied any history of constipation. So, the causative factor in this reported case will remain unknown after the definitive treatment [5].

In pediatric surgical practice volvulus of the sigmoid colon remains a rare occurrence. Only a few isolated case reports and case series have been reported to date in the literature [4]. The median age was 7 years and the male to female ratio was 3.5:1 [6]. Volvulus is more common in males, possibly because the large volume of the female pelvis facilitates spontaneous untwisting.

Two distinct presentations (acute and recurrent) were identified. Abdominal symptoms dominated the clinical picture. The most common symptoms are abdominal pain that is relieved by passage of stool or flatus, abdominal distention, and vomiting [6]. All three symptoms were present in our patient.

The sensitivity of the coffee bean sign for sigmoid volvulus in children is reported to be only 16 to 29% in a review of pediatric cases in the literature [6]. A plain abdominal X-ray is suggestive of sigmoid volvulus in 29% of cases, while barium enema is diagnostic in 61% of cases [7]. While the literature says plain film is not highly sensitive in detecting sigmoid volvulus signs in children, our patient showed a classic finding.

Although, the classic bird’s beak deformity seen on contrast enema is pathognomonic for volvulus, Mellor and Drake described a twisted appearance to be more common [8]. This twist was highly evident in our case in both barium enema studies. Barium enemas either confirmed or were highly suggestive of sigmoid volvulus. Reduction by barium enema was successful in 77% (10 of 13) of the attempts [6].

Treatment for sigmoid volvulus remains controversial in children. If the patient is stable, non-operative reduction of the volvulus with barium enema or sigmoidoscopy may first be attempted [9]. When there are no signs of peritonitis and an endoscopy unit is available with both pediatric and adult expertise, endoscopic decompression and detorsion should be the initial step of treatment in order to relieve symptoms and to prepare the patient for the surgical exploration [10].

The definitive treatment is sigmoidectomy, either with primary anastomosis or colostomy. Recurrence is common when detorsion without resection is performed (operative 25%, non-operative 35%) [6]. The prognosis of definitive treatment of sigmoid volvulus is excellent, provided it is diagnosed early and treated promptly [4].

## Conclusions

Sigmoid volvulus is an uncommon problem in children and adolescents, and is rarely considered a diagnosis in this group as a cause of intestinal obstruction. Hence, pediatric surgeons should maintain a high index of suspicion, in order not to miss them, as any delay in instituting treatment has a devastating effect on morbidity as well as mortality. Early diagnosis and prompt treatment confer an excellent prognosis.
